# *N*-(7-Dibromo­methyl-5-methyl-1,8-naphthyridin-2-yl)acetamide–pyrrolidine-2,5-dione (1/1)

**DOI:** 10.1107/S1600536812051112

**Published:** 2013-01-04

**Authors:** Gao-Zhang Gou, Jun-Feng Kou, Qing-Di Zhou, Shao-Ming Chi

**Affiliations:** a College of Chemistry and Chemical Engineering, Yunnan Normal University, Kunming 650500, People’s Republic of China; b School of Chemistry, The University of Sydney, Sydney, NSW 2006, Australia

## Abstract

In the title co-crystal, C_12_H_11_Br_2_N_3_O·C_4_H_5_NO_2_, the naphthyridine derivative and the pyrrolidine-2,5-dione mol­ecules have crystallographic mirror-plane symmetry with all non-H atoms, except the Br atom, located on the mirror plane. In the crystal, N—H⋯N, N—H⋯O and C—H⋯O hydrogen bonds link the mol­ecules into heterodimers. These dimers are further linked into a one-dimensional structure along [010] by weak C—Br⋯O inter­actions [Br⋯O = 3.028 (5) Å and C—Br⋯O = 158.52 (4)°].

## Related literature

For coordination properties of 1,8-naphthyridine ligands, see: Gan *et al.* (2011 ▶); Chang *et al.* (2011 ▶); Das *et al.* (2012 ▶); Li *et al.* (2011 ▶). For similar structures, see: Li *et al.* (2011 ▶). For applications of similar compounds, see: Samadi *et al.* (2011 ▶); Li *et al.* (2012 ▶); Tanaka *et al.* (2012 ▶). For information on their synthesis, see: Henry & Hammond (1977 ▶); Wang *et al.* (2008 ▶).
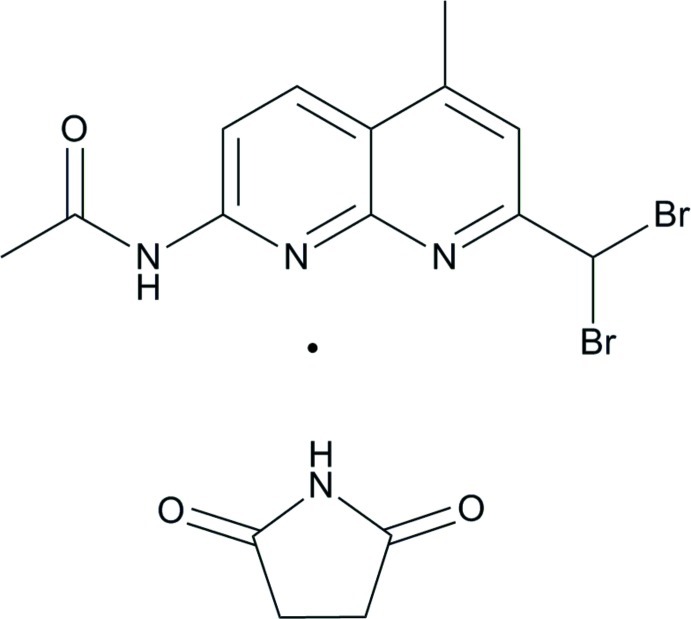


## Experimental

### 

#### Crystal data

C_12_H_11_Br_2_N_3_O·C_4_H_5_NO_2_
*M* *_r_* = 472.15
Monoclinic, 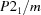

*a* = 11.537 (2) Å
*b* = 7.0093 (14) Å
*c* = 11.632 (2) Å
β = 106.57 (3)°
*V* = 901.6 (3) Å^3^
*Z* = 2
Mo *K*α radiation
μ = 4.52 mm^−1^
*T* = 293 K
0.21 × 0.19 × 0.18 mm

#### Data collection

Rigaku R-AXIS RAPID diffractometer
Absorption correction: multi-scan (*ABSCOR*; Higashi, 1995 ▶) *T*_min_ = 0.450, *T*_max_ = 0.497
7056 measured reflections
1723 independent reflections
1220 reflections with *I* > 2σ(*I*)
*R*_int_ = 0.042

#### Refinement

*R*[*F* ^2^ > 2σ(*F* ^2^)] = 0.051
*wR*(*F* ^2^) = 0.164
*S* = 1.12
1723 reflections
148 parameters
H-atom parameters constrained
Δρ_max_ = 0.72 e Å^−3^
Δρ_min_ = −0.92 e Å^−3^

### 

Data collection: *PROCESS-AUTO* (Rigaku, 1998 ▶); cell refinement: *PROCESS-AUTO*; data reduction: *CrystalClear* (Rigaku/MSC, 2006 ▶); program(s) used to solve structure: *SHELXS97* (Sheldrick, 2008 ▶); program(s) used to refine structure: *SHELXL97* (Sheldrick, 2008 ▶); molecular graphics: *DIAMOND* (Brandenburg,1999 ▶); software used to prepare material for publication: *SHELXL97*.

## Supplementary Material

Crystal structure: contains datablock(s) I, global. DOI: 10.1107/S1600536812051112/gk2541sup1.cif

Structure factors: contains datablock(s) I. DOI: 10.1107/S1600536812051112/gk2541Isup2.hkl

Supplementary material file. DOI: 10.1107/S1600536812051112/gk2541Isup3.cml

Additional supplementary materials:  crystallographic information; 3D view; checkCIF report

## Figures and Tables

**Table 1:** Hydrogen-bond geometry (Å, °)

*D*—H⋯*A*	*D*—H	H⋯*A*	*D*⋯*A*	*D*—H⋯*A*
C6—H6*A*⋯O1	0.93	2.25	2.838 (10)	121
C9—H9*A*⋯O3^i^	0.98	2.55	3.507 (11)	166
N4—H4*A*⋯N1^ii^	0.86	2.56	3.317 (9)	147
N4—H4*A*⋯N2^ii^	0.86	2.24	3.045 (8)	157
N3—H3*A*⋯O2^i^	0.86	2.18	3.038 (9)	177

Symmetry codes: (i) 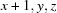; (ii) 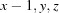.

**Fig. 1.**
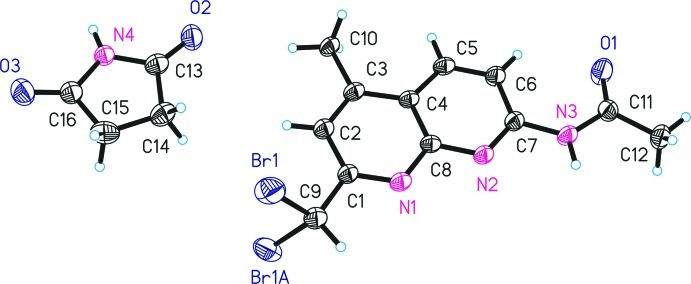
The molecular structure of the title compound with atom labels and 30% probability displacement ellipsoids, and the disorders of hydrogen atoms are shown.

**Fig. 2.**
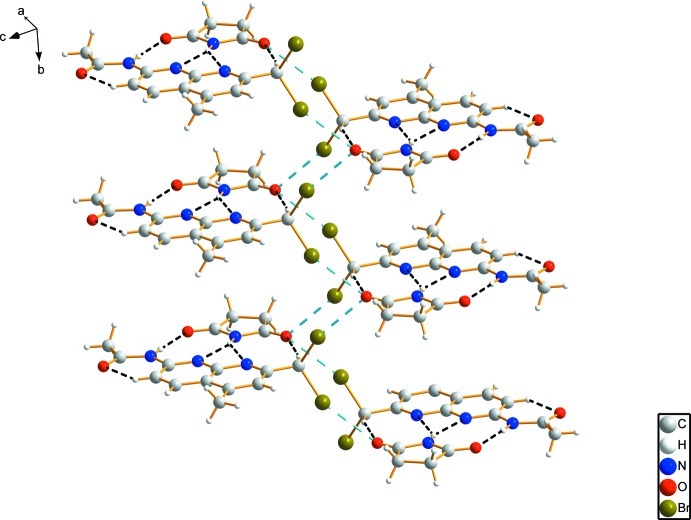
A view of the crystal packing. Hydrogen bonds are shown as black dashed lines, while weak contacts as the blue ones.

## supplementary crystallographic information

### Comment

Structures and chemical properties of 1,8-naphthyridine derivatives have been investigated owing to their interesting complexation properties, medical uses and chemical applications. They can act as the ligands linking to metals *via* several coordination modes (Gan *et al.*, 2011; Chang *et al.*, 2011; Das *et al.*, 2012; Li *et al.*, 2011), as drugs (Samadi *et al.*, 2011) and as molecular probe (Li *et al.*, 2012; Tanaka *et al.*, 2012). Herein we report the synthesis and structure of the title co-crystal which was unintentionally obtained during the synthesis of the naphthiridine derivative.

The structure of the title co-crystal is shown in Figs. 1 and 2 and hydrogen-bond geometry is given in Table 1. Both molecules are located on a mirror plane. There are three (N—H···N, N—H···O and C—H···O) intermoleular hydrogen bonds between the crystal components linking the molecules to form heterodimers. The complementarity of hydrogen-bonding interactions stabilizes the dimeric structure, and, most probably, it is the reason why the two components were not easily separated during chromatographic procedure.

### Experimental

7-Acetylamino-2,4-dimethyl-1,8-naphthyridine (Wang *et al.*, 2008; Henry & Hammond, 1977) (500 mg, 2,32 mmol) and *N*-bromosuccinimide (0.49 g, 2.79 mmol) were added to an acetonitrile (20 ml) solution in the nitrogen atmosphere. The mixture was stirred at room temperature in the presence of light, a 250 W infrared lamp was used as a light source, for 4 hrs. Excess solvent was removed and the crude product was purified by column chromatography using dichloromethane/methanol (39:1) as the mobile phase to give a white powder. Yield: 250 mg (30%). Crystals suitable for X-ray analysis were obtained by slow diffusion of diethyl ether into the solution of the powder in dichloromethane. To remove succinimide from the sample several cycles of purification by column chromatography had to be carried out. The pure white brominated naphthyridine compound was characterized by ^1^H NMR (500 MHz, CDCl_3_): δ=p.p.m. 8.86 (s, 1H NH), 8.59 (d, J = 9.1 Hz, 1H naphthyridyl proton), 8.40 (d, J = 9.1 Hz, 1H naphthyridyl proton), 7.81 (s, 1H naphthyridyl proton), 6.77 (s, 1H CH), 2.79 (s, 3H), 2.32 (s, 3H).

### Refinement

All H atoms were placed in calculated positions. The H atoms were constrained to an ideal geometry with C—H distances of 0.93–0.96 Å, *U*_iso_(H) = 1.5*U*_eq_(C) for methyl H atoms and *U*_iso_(H) =1.2*U*_eq_(C) for the remaining H atoms, and N—H distance of 0.86 Å, *U*_iso_(H) = 1.2*U*_eq_(N).

### Crystal data

**Table:**

C_12_H_11_Br_2_N_3_O·C_4_H_5_NO_2_	*F*(000) = 468
*M**_r_* = 472.15	*D*_x_ = 1.739 Mg m^−^^3^
Monoclinic, *P*2_1_/*m*	Mo *K*α radiation, λ = 0.71073 Å
Hall symbol: -P 2yb	Cell parameters from 25 reflections
*a* = 11.537 (2) Å	θ = 3.4–25.0°
*b* = 7.0093 (14) Å	µ = 4.52 mm^−^^1^
*c* = 11.632 (2) Å	*T* = 293 K
β = 106.57 (3)°	Block, colourless
*V* = 901.6 (3) Å^3^	0.21 × 0.19 × 0.18 mm
*Z* = 2	

### Data collection

**Table:**

Rigaku R-AXIS RAPID diffractometer	1723 independent reflections
Radiation source: fine-focus sealed tube	1220 reflections with *I* > 2σ(*I*)
Graphite monochromator	*R*_int_ = 0.042
ω scan	θ_max_ = 25.0°, θ_min_ = 3.4°
Absorption correction: multi-scan (*ABSCOR*; Higashi, 1995)	*h* = −13→13
*T*_min_ = 0.450, *T*_max_ = 0.497	*k* = −8→7
7056 measured reflections	*l* = −13→13

### Refinement

**Table:**

Refinement on *F*^2^	Primary atom site location: structure-invariant direct methods
Least-squares matrix: full	Secondary atom site location: difference Fourier map
*R*[*F*^2^ > 2σ(*F*^2^)] = 0.051	Hydrogen site location: inferred from neighbouring sites
*wR*(*F*^2^) = 0.164	H-atom parameters constrained
*S* = 1.12	*w* = 1/[σ^2^(*F*_o_^2^) + (0.0757*P*)^2^ + 1.6111*P*] where *P* = (*F*_o_^2^ + 2*F*_c_^2^)/3
1723 reflections	(Δ/σ)_max_ < 0.001
148 parameters	Δρ_max_ = 0.72 e Å^−^^3^
0 restraints	Δρ_min_ = −0.92 e Å^−^^3^

### Special details

**Table:**

Geometry. All e.s.d.'s (except the e.s.d. in the dihedral angle between two l.s. planes) are estimated using the full covariance matrix. The cell e.s.d.'s are taken into account individually in the estimation of e.s.d.'s in distances, angles and torsion angles; correlations between e.s.d.'s in cell parameters are only used when they are defined by crystal symmetry. An approximate (isotropic) treatment of cell e.s.d.'s is used for estimating e.s.d.'s involving l.s. planes.
Refinement. Refinement of *F*^2^ against ALL reflections. The weighted *R*-factor *wR* and goodness of fit *S* are based on *F*^2^, conventional *R*-factors *R* are based on *F*, with *F* set to zero for negative *F*^2^. The threshold expression of *F*^2^ > σ(*F*^2^) is used only for calculating *R*-factors(gt) *etc*. and is not relevant to the choice of reflections for refinement. *R*-factors based on *F*^2^ are statistically about twice as large as those based on *F*, and *R*- factors based on ALL data will be even larger.

### Fractional atomic coordinates and isotropic or equivalent isotropic displacement parameters (Å^2^)

**Table:**

	*x*	*y*	*z*	*U*_iso_*/*U*_eq_	
C1	0.5310 (7)	0.2500	0.7838 (6)	0.0437 (17)	
C2	0.4522 (7)	0.2500	0.8575 (7)	0.0486 (18)	
H2A	0.3689	0.2500	0.8226	0.058*	
C3	0.4977 (7)	0.2500	0.9803 (6)	0.0452 (17)	
C4	0.6257 (7)	0.2500	1.0284 (6)	0.0405 (16)	
C5	0.6882 (7)	0.2500	1.1520 (6)	0.0482 (18)	
H5A	0.6446	0.2500	1.2080	0.058*	
C6	0.8109 (7)	0.2500	1.1903 (7)	0.053 (2)	
H6A	0.8518	0.2500	1.2717	0.063*	
C7	0.8749 (6)	0.2500	1.1028 (6)	0.0456 (18)	
C8	0.6974 (7)	0.2500	0.9487 (6)	0.0415 (16)	
C9	0.4859 (8)	0.2500	0.6506 (7)	0.058 (2)	
H9A	0.5554	0.2500	0.6180	0.069*	
C10	0.4146 (7)	0.2500	1.0597 (7)	0.0491 (18)	
H10A	0.3319	0.2500	1.0112	0.074*	
H10B	0.4298	0.1382	1.1094	0.074*	
C11	1.0841 (7)	0.2500	1.2427 (7)	0.055 (2)	
C12	1.2128 (7)	0.2500	1.2408 (8)	0.067 (2)	
H12A	1.2652	0.2500	1.3214	0.101*	
H12B	1.2279	0.1382	1.1997	0.101*	
C13	0.0377 (8)	0.2500	0.8080 (8)	0.057 (2)	
C14	0.0885 (8)	0.2500	0.7021 (8)	0.069 (2)	
H14A	0.1368	0.1384	0.7023	0.082*	
C15	−0.0232 (9)	0.2500	0.5948 (8)	0.080 (3)	
H15A	−0.0251	0.1387	0.5463	0.095*	
C16	−0.1280 (8)	0.2500	0.6435 (8)	0.056 (2)	
N1	0.6503 (6)	0.2500	0.8271 (5)	0.0477 (15)	
N2	0.8206 (6)	0.2500	0.9860 (5)	0.0461 (15)	
N3	1.0008 (6)	0.2500	1.1320 (5)	0.0524 (16)	
H3A	1.0303	0.2500	1.0719	0.063*	
N4	−0.0857 (6)	0.2500	0.7663 (5)	0.0502 (16)	
H4A	−0.1322	0.2500	0.8124	0.060*	
O1	1.0553 (6)	0.2500	1.3339 (5)	0.093 (3)	
O2	0.0955 (5)	0.2500	0.9136 (5)	0.0759 (19)	
O3	−0.2342 (6)	0.2500	0.5882 (6)	0.081 (2)	
Br1	0.38805 (7)	0.02689 (12)	0.59139 (6)	0.0801 (4)	

### Atomic displacement parameters (Å^2^)

**Table:**

	*U*^11^	*U*^22^	*U*^33^	*U*^12^	*U*^13^	*U*^23^
C1	0.041 (4)	0.052 (4)	0.042 (4)	0.000	0.018 (3)	0.000
C2	0.043 (4)	0.060 (5)	0.045 (4)	0.000	0.018 (3)	0.000
C3	0.045 (4)	0.048 (4)	0.050 (4)	0.000	0.025 (4)	0.000
C4	0.044 (4)	0.044 (4)	0.039 (4)	0.000	0.020 (3)	0.000
C5	0.053 (5)	0.059 (5)	0.041 (4)	0.000	0.026 (3)	0.000
C6	0.046 (5)	0.079 (6)	0.036 (4)	0.000	0.016 (3)	0.000
C7	0.041 (4)	0.054 (4)	0.044 (4)	0.000	0.016 (3)	0.000
C8	0.046 (5)	0.046 (4)	0.035 (4)	0.000	0.018 (3)	0.000
C9	0.054 (5)	0.076 (6)	0.047 (4)	0.000	0.021 (4)	0.000
C10	0.041 (4)	0.062 (5)	0.051 (4)	0.000	0.024 (3)	0.000
C11	0.046 (5)	0.075 (6)	0.041 (4)	0.000	0.009 (4)	0.000
C12	0.042 (5)	0.102 (7)	0.057 (5)	0.000	0.012 (4)	0.000
C13	0.059 (6)	0.061 (5)	0.058 (5)	0.000	0.027 (4)	0.000
C14	0.059 (6)	0.083 (6)	0.074 (6)	0.000	0.034 (5)	0.000
C15	0.091 (8)	0.107 (8)	0.052 (5)	0.000	0.039 (5)	0.000
C16	0.054 (5)	0.061 (5)	0.054 (5)	0.000	0.017 (4)	0.000
N1	0.047 (4)	0.060 (4)	0.043 (3)	0.000	0.024 (3)	0.000
N2	0.042 (4)	0.062 (4)	0.039 (3)	0.000	0.020 (3)	0.000
N3	0.043 (4)	0.079 (5)	0.041 (3)	0.000	0.022 (3)	0.000
N4	0.042 (4)	0.070 (4)	0.042 (3)	0.000	0.017 (3)	0.000
O1	0.051 (4)	0.188 (8)	0.041 (3)	0.000	0.014 (3)	0.000
O2	0.054 (4)	0.114 (6)	0.057 (4)	0.000	0.012 (3)	0.000
O3	0.067 (5)	0.116 (6)	0.057 (4)	0.000	0.011 (3)	0.000
Br1	0.0936 (7)	0.0929 (6)	0.0563 (5)	−0.0237 (4)	0.0254 (4)	−0.0161 (3)

### Geometric parameters (Å, º)

**Table:**

C1—N1	1.324 (9)	C9—H9A	0.9800
C1—C2	1.417 (10)	C10—H10A	0.9600
C1—C9	1.487 (10)	C10—H10B	0.9600
C2—C3	1.373 (10)	C11—O1	1.199 (10)
C2—H2A	0.9300	C11—N3	1.370 (10)
C3—C4	1.422 (10)	C11—C12	1.490 (11)
C3—C10	1.509 (9)	C12—H12A	0.9600
C4—C8	1.407 (10)	C12—H12B	0.9601
C4—C5	1.413 (10)	C13—O2	1.220 (10)
C5—C6	1.357 (11)	C13—N4	1.367 (10)
C5—H5A	0.9300	C13—C14	1.508 (12)
C6—C7	1.418 (10)	C14—C15	1.517 (13)
C6—H6A	0.9300	C14—H14A	0.9600
C7—N2	1.324 (9)	C15—C16	1.474 (13)
C7—N3	1.394 (9)	C15—H15A	0.9600
C8—N1	1.363 (9)	C16—O3	1.210 (10)
C8—N2	1.363 (9)	C16—N4	1.372 (10)
C9—Br1	1.936 (5)	N3—H3A	0.8600
C9—Br1^i^	1.936 (5)	N4—H4A	0.8600
			
N1—C1—C2	123.1 (7)	C3—C10—H10A	109.8
N1—C1—C9	114.4 (6)	C3—C10—H10B	109.3
C2—C1—C9	122.4 (7)	H10A—C10—H10B	109.5
C3—C2—C1	120.5 (7)	O1—C11—N3	122.3 (8)
C3—C2—H2A	119.7	O1—C11—C12	122.8 (7)
C1—C2—H2A	119.7	N3—C11—C12	114.9 (7)
C2—C3—C4	117.1 (6)	C11—C12—H12A	109.8
C2—C3—C10	121.0 (7)	C11—C12—H12B	109.3
C4—C3—C10	121.9 (7)	H12A—C12—H12B	109.5
C8—C4—C5	116.5 (7)	O2—C13—N4	124.9 (8)
C8—C4—C3	118.7 (6)	O2—C13—C14	126.5 (8)
C5—C4—C3	124.9 (6)	N4—C13—C14	108.6 (8)
C6—C5—C4	121.0 (7)	C13—C14—C15	103.6 (7)
C6—C5—H5A	119.5	C13—C14—H14A	111.0
C4—C5—H5A	119.5	C15—C14—H14A	111.0
C5—C6—C7	118.2 (7)	C16—C15—C14	106.4 (7)
C5—C6—H6A	120.9	C16—C15—H15A	110.0
C7—C6—H6A	120.9	C14—C15—H15A	110.8
N2—C7—N3	113.9 (6)	O3—C16—N4	124.0 (8)
N2—C7—C6	123.1 (7)	O3—C16—C15	127.8 (8)
N3—C7—C6	123.1 (7)	N4—C16—C15	108.2 (8)
N1—C8—N2	113.6 (6)	C1—N1—C8	117.3 (6)
N1—C8—C4	123.3 (7)	C7—N2—C8	118.1 (6)
N2—C8—C4	123.1 (6)	C11—N3—C7	129.2 (7)
C1—C9—Br1	111.5 (3)	C11—N3—H3A	115.4
C1—C9—Br1^i^	111.5 (3)	C7—N3—H3A	115.4
Br1—C9—Br1^i^	107.7 (4)	C16—N4—C13	113.2 (7)
C1—C9—H9A	108.7	C16—N4—H4A	123.4
Br1—C9—H9A	108.7	C13—N4—H4A	123.4
Br1^i^—C9—H9A	108.7		

Symmetry code: (i) *x*, −*y*+1/2, *z*.

### Hydrogen-bond geometry (Å, º)

**Table:**

*D*—H···*A*	*D*—H	H···*A*	*D*···*A*	*D*—H···*A*
C6—H6*A*···O1	0.93	2.25	2.838 (10)	121
C9—H9*A*···O3^ii^	0.98	2.55	3.507 (11)	166
N4—H4*A*···N1^iii^	0.86	2.56	3.317 (9)	147
N4—H4*A*···N2^iii^	0.86	2.24	3.045 (8)	157
N3—H3*A*···O2^ii^	0.86	2.18	3.038 (9)	177

Symmetry codes: (ii) *x*+1, *y*, *z*; (iii) *x*−1, *y*, *z*.
